# *Yersinia pestis* Ail: multiple roles of a single protein

**DOI:** 10.3389/fcimb.2012.00103

**Published:** 2012-08-06

**Authors:** Anna M. Kolodziejek, Carolyn J. Hovde, Scott A. Minnich

**Affiliations:** School of Food Science, University of IdahoMoscow, ID, USA

**Keywords:** *Yersinia pestis*, Ail, OmpX, serum resistance, virulence, adhesion, invasion, T3SS

## Abstract

*Yersinia pestis* is one of the most virulent bacteria identified. It is the causative agent of plague—a systemic disease that has claimed millions of human lives throughout history. *Y. pestis* survival in insect and mammalian host species requires fine-tuning to sense and respond to varying environmental cues. Multiple *Y. pestis* attributes participate in this process and contribute to its pathogenicity and highly efficient transmission between hosts. These include factors inherited from its enteric predecessors; *Y. enterocolitica* and *Y. pseudotuberculosis*, as well as phenotypes acquired or lost during *Y. pestis* speciation. Representatives of a large *Enterobacteriaceae* Ail/OmpX/PagC/Lom family of outer membrane proteins (OMPs) are found in the genomes of all pathogenic *Yersiniae*. This review describes the current knowledge regarding the role of Ail in *Y. pestis* pathogenesis and virulence. The pronounced role of Ail in the following areas are discussed (1) inhibition of the bactericidal properties of complement, (2) attachment and *Yersinia* outer proteins (Yop) delivery to host tissue, (3) prevention of PMNL recruitment to the lymph nodes, and (4) inhibition of the inflammatory response. Finally, Ail homologs in *Y. enterocolitica* and *Y. pseudotuberculosis* are compared to illustrate differences that may have contributed to the drastic bacterial lifestyle change that shifted *Y. pestis* from an enteric to a vector-born systemic pathogen.

## *Yersinia pestis*—brief characteristics of a most notorious pathogen

*Y. pestis* is a Gram negative, non-motile, facultative anaerobic rod that exhibits bipolar staining (classic safety-pin pattern). It is a zoonotic pathogen and the causative agent of plague—a systemic disease that has claimed millions of human lives throughout history. The infectious dose is extremely low, estimated between 1 and 10 organisms, making it one of the most virulent bacteria identified (Perry and Fetherston, 1997). In its sylvatic cycle, *Y. pestis* moves between fleas (the arthropod vector) and rodents (reservoir host) with transmission occurring when the vector takes a blood meal. A state of high bacteremia in the mammalian host at the time of the bite is required for a flea infection. Rarely, the disease can be spread through direct contact between infected animals (Wimsatt and Biggins, 2009). Flea-borne *Y. pestis* infection in humans leads to the development of bubonic and septicemic plague that can progress to secondary pneumonic plague (Perry and Fetherston, 1997; Sebbane et al., 2006). This airborne form of the disease can be transmitted directly between humans without the vector involvement, causing primary pneumonic disease. Human-to-human airborne infection is of special importance because of the rapid spread and progression of the disease (short incubation period up to 2–3 days), and very high mortality rates (approaching 100% if untreated) (Perry and Fetherston, 1997). The characteristics of pneumonic plague highlight the sobering potential for *Y. pestis* employment as a biological warfare agent.

Phylogenetic analyses indicate *Y. pestis* evolved relatively recently (within the last 20,000 years) from *Yersinia pseudotuberculosis*, an enteropathogen that causes self-limiting infections (Achtman et al., 1999, 2004; Skurnik et al., 2000). Even though the bacterial genomes shares >90% similarity, the disease transmission, course of the infection, morbidity, and mortality are dramatically different (Bercovier et al., 1980; Eppinger et al., 2007). One of the reasons *Y. pestis* is so efficient is tight expression control of specific genes in response to the host environment. Characteristic host cycling of *Y. pestis* is reflected in global genetic responses in which temperature and calcium ions are key environmental cues the organism senses (Perry and Fetherston, 1997). This is facilitated by virulence phenotypes common with *Y. pseudotuberculosis* and *Y. enterocolitica*, as well as the acquisition of new determinants due to genetic acquisition and genomic entropy (Ben-Gurion and Shafferman, 1981; Perry and Fetherston, 1997; Parkhill et al., 2001; Moran, 2002; Anisimov et al., 2004; Chain et al., 2004, 2006; Darby et al., 2005; Erickson et al., 2006, 2008; Montminy et al., 2006; Sebbane et al., 2006, 2009; Derbise et al., 2007; Lathem et al., 2007; Hinnebusch and Erickson, 2008; Sun et al., 2008).

The Ail/OmpX/PagC/Lom family is a family of outer membrane proteins (OMPs) found in organisms like *Y. enterocolitica* (Ail), *Y. pseudotuberculosis* (Ail), *Salmonella enterica* serovar *typhimurium* (Rck, PagC), or *Escherichia coli* (OmpX). Particular members of the family are responsible for conferring resistance to complement-mediated killing, survival in macrophages, and adhesion and invasion of host cells (Miller et al., 1989a; Bliska and Falkow, 1992; Heffernan et al., 1992; Cirillo et al., 1996). *Y. pestis* Ail protein (also known as OmpX) has been found to be an important virulence factor during plague pathogenesis (Kolodziejek et al., 2010; Hinnebusch et al., 2011). Although our laboratory first designated this protein as OmpX, accordingly to the annotations in the *Y. pesis* KIM genome, due to the subsequent and common use by others of the designation “Ail” we refer to the protein as Ail, rather than OmpX, in this review.

The prominent role of Ail in serum resistance (Kolodziejek et al., 2007, 2010; Bartra et al., 2008), adhesion to and internalization into host cells (Kolodziejek et al., 2007, 2010; Felek and Krukonis, 2009; Tsang et al., 2010), *Yersinia* outer proteins (Yop) delivery (Felek and Krukonis, 2009; Tsang et al., 2010; Yamashita et al., 2011), and inhibition of inflammatory response has been shown (Felek and Krukonis, 2009; Hinnebusch et al., 2011).

This review is a compendium of the current knowledge of the roles of Ail in *Y. pestis* pathogenesis and virulence. Similarities between Ail from *Y. enterocolitica* and *Y. pseudotuberculosis* are shown to illustrate functional similarity and differences between the proteins. The characteristics of the *Y. pestis* strains used in the studies described below are included in Table 1 to help the reader follow the rational for particular strain use.

**Table 1:** **Characteristics and virulence of selected *Y. pestis* strains used in laboratory studies**.

**Strain**	pgm^*^	pCD1^**^	**pMT1**	**pPCP**	**Infection route**	**LD_50_ mice (References)**	**LD_50_ rats (References)**
*Y. pestis CO92*	+	+	+	+	i.n.	2×10^4^ (Airhart et al., 2008)	1.2×10^3^ (Kolodziejek et al., 2010)
					s.c.	1.7 (Welkos et al., 1997)	<50 (Hinnebusch et al., 2011)
*Y. pestis KIM5*	−	+	+	+	s.c.	>10^7^ (Une and Brubaker, 1984)	–
					i.v.	15 (Une and Brubaker, 1984)	–
*Y. pestis KIM6*	−	−	+	+	s.c.	>10^7^ (Une and Brubaker, 1984)	–
					i.v.	>10^7^ (Une and Brubaker, 1984)	–
*Y. pestis KIM6^+^*	+	−	+	+	s.c.	>10^7^ (Une and Brubaker, 1984)	–
					i.v.	>10^7^ (Une and Brubaker, 1984)	–

* , biofilm-related loci;

** , encodes Type Three Secretory System (T3SS). i.n., intranasal; s.c., subcutaneous; i.v., intravenous.

## Primary, secondary, and tertiary structure of Ail

**Figure 1:**
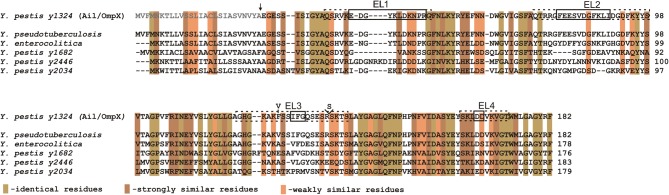
**Multiple strain sequence alignment and amino acid analysis of Ail.** Gray letters indicate the signal sequence and the arrow indicates the signal sequence cleavage site (Kolodziejek et al., 2007). EL1, EL2, EL3, and EL4 designate extracellular loops; dotted line boxes indicate extracellular regions, including just the residues connecting two β strands (solid line boxes), as predicted from the crystal structure by Yamashita et al. (2011). Amino acid variation in the Ail polypeptide among *Y. pestis* strains is indicated by the presence of Val in the position 126 (*Y. pestis* Pestoides F, Angola, *Y. pestis* subsp. *caucasica, altaica, hissarica*) instead of Phe and additional substitution of Ser in the position 137 (*Y. pestis* Pestoides F, *Y. pestis* subsp. *caucasica*) (Eroshenko et al., 2010). Alignments of Ail from *Y. pestis* (*y1324*) with Ail from *Y. pseudotuberculosis* IP 32953, *Y. enterocolitica* 8081c, and three other *Y. pestis* Ail homologs (*y1682, y2446*, and *y2034*) are depicted. Indicated colors represent identical, strongly, and weakly similar residues. Alignments were produced with ClustalW.

The genome of *Y. pestis* KIM encodes four homologs of the Ail protein: *y1324, y1682, y2034*, and *y2446* (designated *YPO2905, YPO2506, YPO2190, YPO1860*, accordingly in *Y. pestis* CO92 genome), which are also present in *Y. pseudotuberculosis* genome. The gene designated *y1324* encodes a protein with a predicted molecular mass of 17.47 kDa (without the signal sequence) and shares the highest amino acid sequence similarity to an Ail protein from *Y. enterocolitica* (~70%) (Kolodziejek et al., 2007; Bartra et al., 2008) (Figure 1).

Analysis of the *ail* nucleotide sequence reveals differences among the *Y. pestis* isolates that result in amino acid variation in the polypeptide chain (Eroshenko et al., 2010). Two variable sites in the gene can be identified: (1) a missense mutation in position 376 that results in a codon change from GTT to TTT and consequently a substitution of Val126 (*Y. pestis* Pestoides F, Angola, *Y. pestis* subsp. *caucasica, altaica, hissarica*) to Phe (*Y. pestis* KIM, CO92, Antiqua, Nepal 516, *Y. pestis* subsp. *ulegeica*) (2) insertion of an AGT triplet at position 406 that results in the addition of an extra Ser137 in the Ail polypeptide chain (*Y. pestis* Pestoides F, *Y. pestis* subsp. *caucasica*) (Figure 1).

Bioinformatic analysis of the *y1324* gene also reveals two potential products differing in the length of the signal sequence peptide (predicted 38 and 26-residues long). The exact translational start sites have not been confirmed, but due to the lack of a clear ribosomal binding site and less preferable GTG codon start for the 38-residue long signal sequence peptide, the translational start resulting in the shorter signal sequence is preferable. The signal peptide cleavage site is located between Ala and Glu and has been identified by both bioinformatic and peptide ion mapping analysis (Kolodziejek et al., 2007) (Figure 1).

**Figure 2:**
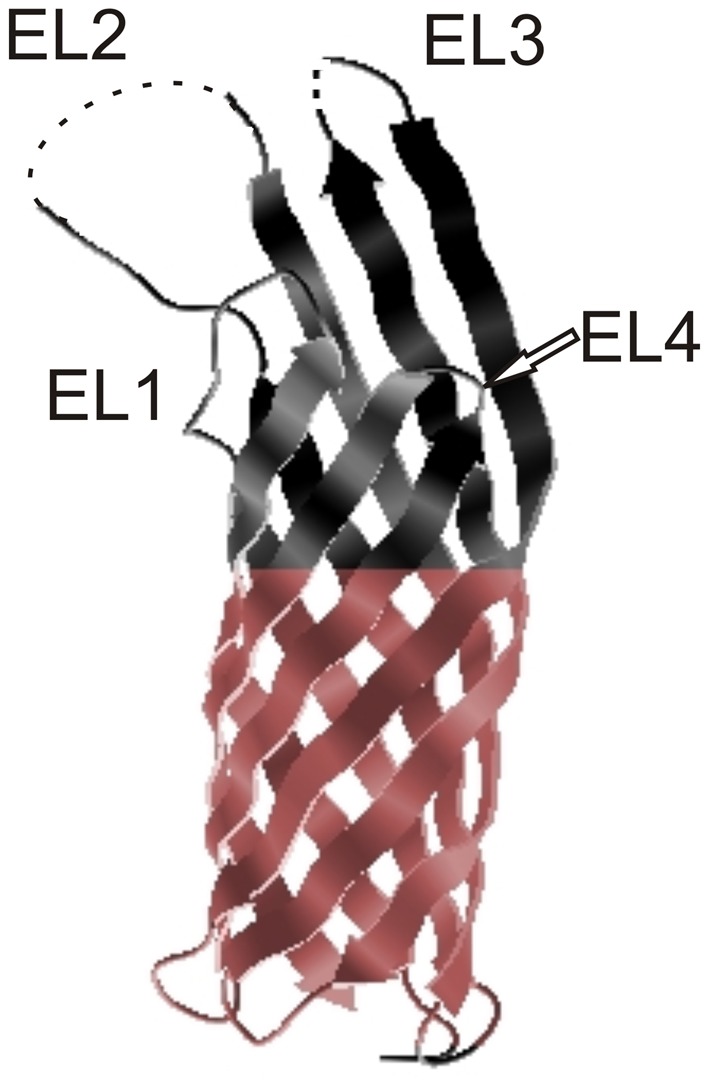
**Crystal structure of Ail.** Extracellular loops are indicated EL1, EL2, EL3, and EL4 and the hydrophobic belt is indicated in color. The tips of EL2 and EL3 are disordered and these regions are marked with dotted lines (Yamashita et al., 2011). The figure was generated from the PDBe Protein Data Bank in Europe.

Ail is a small, monomeric, surface-associated protein localized in the OM (Pieper et al., 2009a). The crystal structure of *Yersinia pestis* Ail is available (Figure 2). It reveals that Ail forms an eight-stranded antiparallel β-barrel with four extracellular loops (Figure 2). The amino acid similarity to the members of the Ail/Lom/OmpX family is the highest for the transmembrane regions with little sequence conservation in the extracellular loops (Bartra et al., 2008; Yamashita et al., 2011). No obvious channel through the barrel in the tertiary model can be found (Yamashita et al., 2011). A hydrophobic cleft and two regions with positive charge found on the extracellular surface are indicated to participate in Ail-conferred adhesion to substrates (Yamashita et al., 2011). The NMR and CD (circular dichroism) structural studies also confirm that Ail adopts a transmembrane β-barrel in micells and lipid bilayers (Plesniak et al., 2011).

## Regulation of Ail expression

Ail is the most highly transcribed gene in the entire *Y. pestis* transcriptome (Chauvaux et al., 2007, 2011) and the protein encompasses ~20–30% of the total OM proteome when the bacterium is grown at 37°C, the optimal temperature for Ail expression (Myers-Morales et al., 2007; Pieper et al., 2009a,b). The protein is still abundant, but expressed at lower levels at ambient temperature (28°C) (Kolodziejek et al., 2007; Bartra et al., 2008; Pieper et al., 2009a,b) and the expression is minimal at 6°C (Bartra et al., 2008). The label-free comparative proteome strategy used to identify new virulence targets reveals that Ail clusters together with plasmid pCD1 virulence-associated proteins; the protein displays a similar pattern of expression and abundance shown for YopD, a secreted effector protein that is part of the type three secretion system (T3SS) (Hixson et al., 2006). Transcription of Ail can be also altered due to the mutagenesis of other OM components. Studies show that Ail is down-regulated at 26°C, but not at 37°C, when Braun lipoprotein is deleted (Galindo et al., 2010).

Significant presence of Ail in *Y. pestis* OM at ambient temperature is in contrast to Ail expression in *Y. enterocolitica* in which the protein is expressed at very low levels at this temperature (Bliska and Falkow, 1992; Pierson and Falkow, 1993). Additionally, the overall comparison of the *ail* transcriptional pattern in *Y. pestis* and *Y. pseudotuberculosis* reveals that the expression of *ail* in *Y. pestis* is at a much higher level than in *Y. pseudotuberculosis* (Chauvaux et al., 2011). This implies that the two organism, despite the almost identical gene sequence, use the Ail in a different manner depending on the physiological requirements and this could potentially explain some differences in pathogenicity between the two (Chauvaux et al., 2011).

## Ail confers serum resistance in *Y. pestis*

Complement-dependent bacterial killing is one of the first lines of innate immunity against pathogens (Tedesco, 2008). Thus, resistance to serum complement is an essential phenotype for bacterial survival in blood. *Y. pestis* transmission between insect and mammalian species is determined by continual contact of the bacteria with blood components (Perry and Fetherston, 1997). *Y. pestis* can resist the bactericidal effects of serum when it is grown at 26°C and 37°C, but not at 6°C (Anisimov et al., 2005). These temperatures correlate with ambient, mammalian core body, and insect hibernation conditions, respectively (Anisimov et al., 2005); and complement resistance reflects Ail expression pattern (Bartra et al., 2008). Out of four Ail/OmpX homologs encoded in the *Y. pestis* genome, only *y1324* confers resistance to human serum. Deletion of this gene results in a rapid, essentially 100% loss of serum resistance (Kolodziejek et al., 2007; Bartra et al., 2008). This loss is attributed to the action of complement because heat-inactivated serum does not have lethal properties (Kolodziejek et al., 2007; Bartra et al., 2008). Additionally, the *ail* gene confers resistance to complement killing by rat, goat, sheep, rabbit, and guinea pig sera (Bartra et al., 2008; Kolodziejek et al., 2010). Interestingly, complement-killing of the Ail deletion mutant is not observed when the bacteria are incubated with mouse sera (Bartra et al., 2008; Kolodziejek et al., 2010); phenomenon observed with a number of common microorganisms that can be killed by sera of other species, but remain resistant to murine sera (Marcus et al., 1954; Kolodziejek et al., 2010). The limited bactericidal activity of murine sera remains unclear.

**Figure 3:**
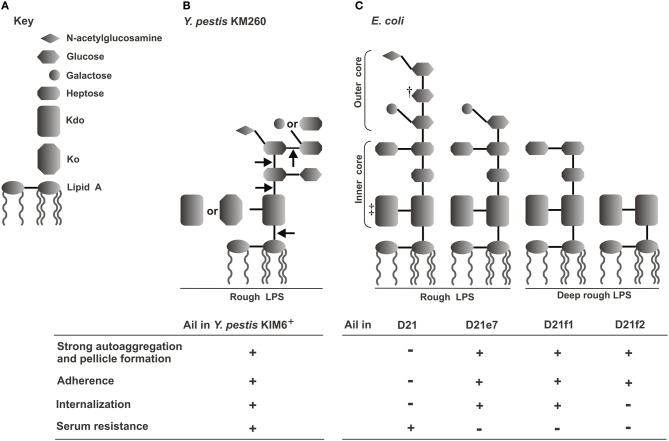
**Correlation of *E. coli* and *Y. pestis* LPS structures and Ail-mediated phenotypes—schematic representation (Kolodziejek et al., 2010). (A)** Key to LPS moieties. **(B)** LPS core structure of *Y. pestis* KM260 grown at ambient temperature; arrows indicate truncation of the core residues that resulted in loss of serum resistance (Knirel et al., 2007; Dentovskaya et al., 2011). **(C)** LPS structures of *E. coli* K-12 D21 and its isogenic mutants (Yu and Mizushima, 1982; Nikaido and Vaara, 1985; Razatos et al., 1998; Junkes et al., 2008; Prokhorenko et al., 2009). ^†^, some sources indicate galactose (Junkes et al., 2008); ^‡^, some sources indicate two or three KDO residues (Nikaido and Vaara, 1985). The table shows presence (+) or lack (−) of Ail-mediated phenotypes (autoaggregation and pellicle formation, adhesion to Hep-2 cells, internalization into Hep-2 cells, and human serum resistance) related to a particular strain (Kolodziejek et al., 2007, 2010).

Ail expressed in a bacterial host other than *Y. pestis* (e.g., *E. coli*) confers serum resistance independently from other *Y. pestis* proteins or LPS (Bartra et al., 2008; Kolodziejek et al., 2010). However, serum resistance does depend on the LPS core structure of the organism in which Ail is being expressed (Figure 3). This can be inferred from indirect and direct evidence. The indirect evidence comes from the observation that *Y. pestis* strains that have shortened LPS core, through directed mutagenesis or as a results of spontaneous mutations, have altered sensitivity to human serum (Anisimov et al., 2005; Knirel et al., 2006, 2007; Dentovskaya et al., 2011) (Figure 3B). *Y. pestis* full-length LPS inner core, composed of six residues, is required for resistance to human complement, while the outer core monosaccharides do not play a critical role (Dentovskaya et al., 2011). The direct evidence is from the study employing a series of isogenic Ail-expressing *E. coli* mutants each with progressively truncated LPS (Figure 3C). Both the inner and outer core are required for effective resistance to the bactericidal effect of complement in *E. coli* as only Ail-expressing *E. coli* with the full core length are resistance to complement (Kolodziejek et al., 2010).

The observed amino acid variation in the Ail polypeptide chain between different *Y. pestis* isolates (see “Primary, secondary, and tertiary structure of Ail” section) seems to be insignificant for the mediation of serum resistance, as all strains were similarly resistant to normal rabbit serum (Eroshenko et al., 2010).

The protection of *Y. pestis* by Ail at either 28 or 37°C contrasts with *Y. enterocolitica* Ail, which confers this trait only at 37°C, reflecting temperature regulation of Ail expression in that organism (Bliska and Falkow, 1992; Pierson and Falkow, 1993). It has been shown that Ail in *Y. pseudotuberculosis* also confers serum resistance when bacteria are grown at 37°C (Yang et al., 1996). In *Y. enterocolitica* and *Y. pseudotuberculosis* three factors contribute to serum resistance: Ail, YadA, and O-chain in LPS (Wachter and Brade, 1989; Bliska and Falkow, 1992; Pierson and Falkow, 1993; Yang et al., 1996; Biedzka-Sarek et al., 2005; Heise and Dersch, 2006). *Y. pestis* does not express YadA or O-chain due to mutations inactivating both the *yadA* and several genes in the O-chain biosynthetic gene cluster (Rosqvist et al., 1988; Skurnik et al., 2000; Prior et al., 2001); thus they cannot play any role in *Y. pestis* serum resistance. Hence, acquisition of Ail-mediated serum resistance at lower temperatures might have been a necessity for the passage of bacteria between mammalian and flea hosts. Although no studies on the mechanisms of serum resistance conferred by *Y. pestis* Ail have been published yet, based on the literature on Ail from *Y. enterocolitica* and *Y. pseudotuberculosis* it is plausible that the protein binds negative regulators of alternative (factor H), and classical and lectin [C4b-binding protein (C4BP)] complement pathways thus preventing complement attack (Biedzka-Sarek et al., 2008a,b; Kirjavainen et al., 2008; Ho et al., 2012).

## Role of Ail in *Y. pestis* adhesion to mammalian cells

Adherence to host tissues is an important infection-determining factor used by many bacterial pathogens (Reid and Sobel, 1987). The most favorable conditions for *Y. pestis* adherence to host cells *in vitro* is pre-incubation of bacteria at ≤28°C in neutral pH (Felek et al., 2010). Of three major adhesins found among the enteropathogenic *Yersiniae*, two, YadA and Invasin, are not produced by *Y. pestis* due to pseudogenes (Rosqvist et al., 1988; Simonet et al., 1996; Forman et al., 2008). PsaA (pH 6 antigen) (Yang et al., 1996; Liu et al., 2006; Felek et al., 2010) and YapE (Lawrenz et al., 2009) are common for *Y. pseudotuberculosis* and *Y. pestis*, have adhesive properties, and their role in adherence was shown in *Y. pseudotuberculosis* (PsaA) and *Y. pestis* (PsaA and YapE). However, a series of studies now show that the primary adhesin in *Y. pestis* is Ail. Deletion of Ail greatly reduces binding of the bacterium to epithelial (HEp-2) and monocytic (THP-1) cells (Kolodziejek et al., 2007; Felek and Krukonis, 2009). Moreover, Ail-expressing *E. coli* facilitates binding to both cell lines, independent from other *Y. pestis* components (Felek and Krukonis, 2009; Kolodziejek et al., 2010); but, similarly to serum resistance, this phenotype depends on the LPS background in which Ail is expressed (Kolodziejek et al., 2010) (Figure 3C). Recent findings indicate that the 3rd extracellular loop of Ail is important for Ail-mediated binding to host cells (Tsang et al., 2012). Ail-conferred adhesiveness correlates with the bacterial autoaggregation, another phenotype mediated by the protein (see section “Ail and autoaggregation of *Y. pestis*” below) (Kolodziejek et al., 2007, 2010).

Most importantly, binding of *Y. pestis* to host cells via Ail, promotes Yop delivery into the primary target of T3SS—the phagocytic cells, as well as into epithelial cells (Felek and Krukonis, 2009; Yamashita et al., 2011). Pretreatment of host cells (HEp-2) with anti-Ail serum and deletion of Ail in *Y. pestis* KIM5 impair Yop delivery, a step critical for *Y. pestis* pathogenesis (Felek and Krukonis, 2009; Yamashita et al., 2011). The significant delay in Yop delivery by an *ail*-negative mutant is observed in both cell lines (HEp-2 and THP-1), particularly obvious after the first 2 h of infection. Around 90% of *Y. pestis* KIM5-infected cells indicate cytotoxic characteristic due to the delivery of Yops vs. only 5–10% of the cells infected with the *ail*-negative strain. However, after 8 h of the infection this difference diminishes to only ~15%, which indicates that *ail*-negative strain is not completely devoid of Yop delivery capabilities (Felek and Krukonis, 2009).

Noteworthy, *Y. pestis* Ail shares functional homology in adherence with Ail in *Y. enterocolitica*, but not with the Ail homolog of *Y. pseudotuberculosis* (Bliska and Falkow, 1992; Yang et al., 1996). Functional discrepancies between Ail from *Y. pestis* and *Y. pseudotuberculosis* call for further investigation to establish that several amino acid substitutions can lead to the dramatic change in function and if other species-specific factors, like LPS, influence the functional differences. In contrast to *Y. enterocolitica* Ail that confers adhesion when bacteria are grown at 37°C (Bliska and Falkow, 1992), Ail in *Y. pestis* can confer this phenotype when grown at both 28 and 37°C (Kolodziejek et al., 2007; Felek et al., 2010).

## Ail interactions with the components of extracellular matrix (ECM)

Ail-mediated binding of *Y. pestis* or *E. coli* to the regions between the host cells during *in vitro* cell culturing indicates that the protein also interacts with the components of the ECM (Kolodziejek et al., 2007; Felek and Krukonis, 2009; Tsang et al., 2010; Yamashita et al., 2011). Purified Ail and Ail-expressing *E. coli* bind specifically to laminin, fibronectin, negatively charged HSPGs, but not collagen I or IV (Tsang et al., 2010; Yamashita et al., 2011). *In vitro* models employing human cell lines and *Y. pestis* show that the bacterium binds to fibronectin and laminin via Ail (Tsang et al., 2010; Yamashita et al., 2011); however, Ail is not the only protein that participates in binding of fibronectin by *Y. pestis* (Tsang et al., 2010). These interactions are responsible for *Y. pestis* attachment to host cells (see above in section “Role of Ail in adhesiveness of *Y. pestis* mammalian cells”). Structural and adhesion (employing purified Ail and Ail-expressing *E. coli*) studies, show that Ail specifically binds a 40 kDA laminin fragment called LG4-5 (Laminin G-like domains 4 and 5) although other laminin domains can also facilitate the adhesion process (Yamashita et al., 2011). Recent studies by Tsang et al. identified that Ail mediates bacterial binding to the centrally located 120 kDA fragment of fibronectin, more precisely to ^9^FNIII module neighboring the RGD site present in ^10^FNIII region (Tsang et al., 2012).

Disruption of Ail interaction with both laminin (Yamashita et al., 2011) and fibronectin (Tsang et al., 2010; Yamashita et al., 2011) alleviate Yop delivery into mammalian cells. Even though Ail interaction with laminin is stronger than with fibronectin in the abiotic models (protein coated glass), it is the binding to fibronectin that contributes to increased Yop delivery into the host cells (HEp-2). The pretreatment of the host cells with anti-laminin antibodies itself does not inhibit Yop delivery; however, a combination of both antibodies, anti-fibronectin, and anti-laminin have a synergistic effect and significantly decreases cytotoxicity compared to treatment with anti-fibronectin antibody alone (Yamashita et al., 2011).

It was also suggested that Ail-mediated interaction of *Y. pestis* with epithelial cells involve heparin sulfate proteoglycan (Zhang et al., 2008). X-ray diffraction crystal structure studies revealed two binding sites for sucrose octasulfate (SOS), a smaller heparin analog, that corresponds to two positively charged sites found on the Ail molecule (Yamashita et al., 2011), designated HBS-I and HBS-II (heparin-binding site 1 and 2, respectively). The HBS-I is the primary binding site for SOS and is located on the extracellular surface on the outside of the β-barrel. Extracellular loops 2 and 3 contribute residues to form HBS-I while extracellular loop 1 contributes to form HBS-II. Mutations in these regions show weak binding to heparin (Yamashita et al., 2011). No studies on the role of Ail-HPSG interactions and delivery of Yop have been done.

## Role of Ail in invasiveness of *Y. pestis* into non-professional phagocytic cells

Bacterial invasiveness is another mechanism to circumvent host immune response (Hess et al., 2001). *Y. pestis* is a facultative intracellular pathogen that resides both inside and outside of the host cells during infection (Perry and Fetherston, 1997). Although macrophages and dendritic cells are the target host cells for *Y. pestis* (Marketon et al., 2005), internalization into non-professional phagocytes occurs (Cowan et al., 2000). Work by Cowan et al. found that *Y. pestis* is highly invasive in HeLa cells when grown at 26, but not at 37°C (Cowan et al., 2000). Deletion of Ail results in impaired internalization of *Y. pestis* (~60-fold decrease) by human epithelial cells (HEp-2) (Kolodziejek et al., 2007), although the invasive properties of *y1324* were not confirmed by Felek et al. (Felek and Krukonis, 2009). When expressed in *E. coli*, Ail confers internalization independently from other *Y. pestis* proteins or LPS, but the efficiency of internalization depends on the LPS core background in which the protein is expressed. Progressive truncation of LPS core results in progressive decrease in Ail-conferred internalization of *E. coli* (Figure 3C). The most pronounced effect is seen with the Ail-expressing deep rough strain that can readily attach to the epithelial cells, but cannot invade them (Kolodziejek et al., 2010).

*Y. pestis* Ail shares functional homology in invasiveness with Ail from *Y. enterocolitica* (Miller and Falkow, 1988) but not with Ail from *Y. pseudotuberculosis* (Yang et al., 1996) (for discussion of the functional discrepancies see “Role of Ail in *Y. pestis* adhesion to mammalian cells”). In contrast to enteropathogenic *Yersiniae* that posses in their repertoire other proteins involved in cellular uptake (invasin and YadA) (Isberg et al., 1987; Eitel and Dersch, 2002), *ail* is the only gene that is both common within the genus and shown to be involved in internalization of *Y. pestis* (while the Pla protease-encoding plasmid pPCP1 was shown to mediate *Y. pestis* internalization (Cowan et al., 2000), it is not present in other *Yersiniae*). YadA structural homolog, YadBC was found to be moderately involved in *Y. pestis* uptake, but its role in *Y. pseudotuberculosis* is not known and the gene is not present in *Y. enterocolitica* (Forman et al., 2008).

## Ail and autoaggregation of *Y. pestis*

The autoaggregation phenotype is often attributed to biofilm formation on abiotic and biotic surfaces in many bacteria and is associated with circumvention of the host defense mechanisms during infection. The autoaggregation phenotype expressed by pellicle formation at the air-liquid interface and by flocculent growth is characteristic for *Y. pestis* and is connected to virulence (Laird and Cavanaugh, 1980; Kolodziejek et al., 2010). It can be observed at 28 and 37°C (Kolodziejek et al., 2007), but not at 6°C (author's observation) which correlates nicely with the pattern of Ail expression (Bartra et al., 2008). Indeed, deletion of Ail confers loss of these characteristics when the bacterium is grown in LB medium (Kolodziejek et al., 2007). Ail-induced autoaggregation does not contribute to serum resistance (by making the bacterial cells inaccessible to complement components), but it is critical for effective bacterial adherence (Kolodziejek et al., 2007, 2010). *Y. pestis ail*-negative mutants that have lost the autoaggregation phenotype and *E. coli* expressing Ail, but showing no flocculent growth, have impaired ability to adhere to epithelial cells (Kolodziejek et al., 2010). Also, the Ail-conferred autoaggregation seems to depend on the display of the protein in a specific LPS core background. A series of Ail-expressing *E. coli* with progressively truncated LPS differ in flocculent growth and pellicle formation (Figure 3) (Kolodziejek et al., 2010).

## *Y. pestis* Ail is a virulence factor

Although Ail in *Y. enterocolitica* is present in all pathogenic strains (Miller et al., 1989b), no changes in virulence are observed when the gene is deleted (Wachtel and Miller, 1995). No information regarding the role of Ail in *Y. pseudotuberculosis* virulence is available. In contrast, Ail in *Y. pestis* is a required virulence factor. Full assessment of Ail's role in *Y. pestis* pathogenesis requires use of multiple animal models. Because murine serum is not bactericidal for *ail*-negative *Y. pestis*, experiments with mice cannot fully assess the role of serum resistance in plague pathogenesis and studies in mice mostly reflect the role of adhesive/invasive phenotype of Ail. Rats make a better model of *Y. pestis* pathogenesis since their serum is similar to humans in that it is bactericidal to the *ail* mutant. Comparison of the mortality between the mice and rats following *Y. pestis* challenge (see below) indicates that serum resistance contributes to virulence more prominently than bacterial association with the host cells. Other models, employing *Caenorhabditis elegans* and fleas, have investigated the role of Ail in virulence and flea transmission.

### Rat model of plague

Ail is an essential virulence factor in the pneumonic (intranasal infection) and bubonic (intradermal injection) model of plague in rats (Kolodziejek et al., 2010; Hinnebusch et al., 2011). Deletion of Ail completely attenuates virulence of *Y. pestis* CO92 in pneumonic model of infection; all rats challenged with the highest dose of 1 × 10^8^ CFU survive, vs. the LD_50_ of 1.2 × 10^3^ CFU established for the wild-type strain (Kolodziejek et al., 2010). Similarly, in a bubonic model of plague infection with up to 3 × 10^4^ CFU of the *Y. pestis* CO92 *ail*-negative strain produce no mortality and no signs of systemic plague morbidity, and most of the animals have no *Y. pestis* cultured from their spleens 17–20 days after the infection (Hinnebusch et al., 2011). These observations are in contrast with animals infected with the wild-type strain: a very small LD_50_ dose (Table 1) leads to death of the animals within 2–5 days post injection. The presence of high wild-type bacterial numbers in the spleen in late stages of infection coupled with systemic signs of terminal disease likewise contrasts with the infection pattern of *ail* mutant. Interestingly, even though the animals infected with the *ail* mutant do not show other signs of morbidity (the animals remain alert and active) they present enlarged lymph nodes that develop during the second week post infection. The purulent lymphadenitis is never observed in the rats infected with the wild-type in which the buboes are characterized with (1) massive accumulation of bacteria within 3–5 days, (2) a hemorrhage, and (3) no signs of pus or strong PMNL response. Meanwhile, *ail*-negative strain develops buboes with the pronounced number of PMNLs; (1) large abscesses encapsulating PMNLs and bacteria, (2) sterile and resolving abscess's with PMNLs, fibroblasts, macrophages, and multinucleated giant cells, and (3) immunoglobulin-producing plasma cells. All these features are evidence of the chronic inflammatory response induced by infections with the *ail* mutant that is not seen with the wild-type (Hinnebusch et al., 2011).

### Mouse model of plague

In mouse models of pneumonic plague, deletion of Ail in *Y. pestis* CO92 extends the time-to-death, but does not alter the LD_50_ value (Table 1). Overall, deletion of Ail delays 100% mortality rate by 24–48 h depending on the challenge dose (10 LD_50_ and 100 LD_50_, respectively) (Kolodziejek et al., 2010). In contrast, intradermal and intravenous (tail vein) infection with *ail* mutants (in *Y. pestis* CO92 and *Y. pestis* KIM5, respectively) attenuates both strains and results in ~10^3^-fold increase in LD_50_ value (Table 1) (Felek and Krukonis, 2009; Hinnebusch et al., 2011). The same KIM5 *ail*-negative strain inoculated via retro-orbital route has no defect in virulence (Bartra et al., 2008). The histopathological changes specific for mice infected with the *ail* mutant include: (1) enlarged lymph nodes containing abscesses and some puss (bubonic model) (Hinnebusch et al., 2011); (2) increase in immune cell infiltration in spleen and liver tissues, their damage, and spleen enlargement (septicemic model) (Felek and Krukonis, 2009). Additionally, different kinetics of bacterial colonization are found for the *ail*-negative strain in the intravenous infection model. At the beginning of infection both mutant and wild-type strains colonizes spleen, liver, and lung tissue similarly, but as the infection progresses, the number of the *ail* mutant bacteria retrieved from the tissues dramatically decline (Felek and Krukonis, 2009).

### *Caenorhabditis elegans* and a flea model of *Y. pestis* infection

Ail is required for biofilm-independent killing of *Caenorhabditis elegans* (Bartra et al., 2008) as its deletion increases survival of adult worms infected with *Y. pestis* KIM5. However, even though deletion of *ail* changes the autoaggregation phenotype in *Y. pestis*, it does not affect biofilm-dependent killing of the nematode (Bartra et al., 2008). Similarly, deletion of *ail* does not alter either the infection rate or bacterial blockage of the flea (*X. cheopsis*) necessary for effective plague transmission from a vector to a mammalian host (Bartra et al., 2008).

## Ail as a potential vaccine candidate against *Y. pestis* human infections

Presently, there is no effective plague vaccine (Williamson, 2009). Killed whole cells vaccines (known since late 1890's) and a live attenuated *Y. pestis* EV76 strain decrease the incidence but do not provide long-lasting efficacious protection against bubonic plague and provide no protection against the pneumonic form of the disease. Additionally, severe side-effects can occur after vaccination. Therefore, the use of the killed whole cells vaccine was discontinued in the United States in 1998 and at present no vaccine is available (Butler, 2009). With the biological threat and appearance of drug resistant strains of *Y. pestis*, there have been increased efforts to develop more effective vaccines to ensure full protection especially against the aerosol-borne form of the disease (Williamson, 2001, 2009). The surface-exposed localization of Ail, the robust induction of inflammatory response in lymphoid tissues caused by *ail*-negative strain, and Ail's overall pronounced role in virulence of this notorious pathogen make the protein a good potential vaccine target. It has already been shown that rats subcutaneously infected with the *ail* mutant have very high serum antibody titers against Caf1 protein, indicative of a strong protective response against *Y. pestis*. Moreover, the persistent infection in the lymph nodes uniquely generated by *ail* mutant in rats, and not by other *Y. pestis* attenuated strains due to their rapid clearance, can serve as an important tool in identifying key players of *Y. pestis* infection and the potential elucidation of new vaccine targets (Hinnebusch et al., 2011).

## Concluding remarks

The elements of *Y. pestis* pathogenesis are still being elucidated. This review describes the current understanding of the role of Ail protein in the course of infection. The explanations behind its pronounced role are provided; (1) inhibition of bactericidal properties of the complement, (2) attachment and Yop delivery to host tissue, (3) prevention of PMNL recruitment to the lymph nodes, (4) inhibition of inflammatory response (Kolodziejek et al., 2007; Felek and Krukonis, 2009; Hinnebusch et al., 2011) are the crucial phenotypes mediated by Ail. Also, some comparison to Ail phenotype in *Y. enterocolitica* and *Y. pseudotuberculosis* is made to illustrate differences that could potentially contributed to the drastic change of the bacterial lifestyle that resulted in a shift from an entero- to a vector-borne systemic pathogen.

### Conflict of interest statement

The authors declare that the research was conducted in the absence of any commercial or financial relationships that could be construed as a potential conflict of interest.
